# Influence of deoxynivalenol-contaminated feed on the immune response of pigs after PRRSV vaccination and infection

**DOI:** 10.1007/s00204-023-03449-9

**Published:** 2023-02-13

**Authors:** Alix Pierron, Eleni Vatzia, Maria Stadler, Kerstin H. Mair, Selma Schmidt, Melissa R. Stas, Sophie Dürlinger, Heinrich Kreutzmann, Christian Knecht, Gyula Balka, Julia Lagler, Marianne Zaruba, Till Rümenapf, Armin Saalmüller, Elisabeth Mayer, Andrea Ladinig, Wilhelm Gerner

**Affiliations:** 1 Institute of Immunology, Department of Pathobiology, University of Veterinary Medicine, Vienna, Austria; 2 University Clinic for Swine, Department for Farm Animals and Veterinary Public Health, University of Veterinary Medicine, Vienna, Austria; 3 Department of Pathology, University of Veterinary Medicine, Budapest, Hungary; 4 Institute of Virology, Department of Pathobiology, University of Veterinary Medicine, Vienna, Austria; 5 DSM-BIOMIN Research Center, Tulln, Austria; 6 Present Address: ENVT (National Veterinary School of Toulouse), IHAP, Toulouse, France; 7 Present Address: The Pirbright Institute, Woking, UK; 8 Present Address: Institute of Virology, University of Zurich, Zurich, Switzerland; 9 National Laboratory of Infectious Animal Diseases, Antimicrobial Resistance, Veterinary Public Health and Food Chain Safety, University of Veterinary Medicine, Budapest, Hungary

**Keywords:** Deoxynivalenol, Pig, PRRSV, Vaccination, Infection, CD4^+^ T cells

## Abstract

The impact of the *Fusarium* mycotoxin deoxynivalenol (DON) on the immune response against porcine reproductive and respiratory syndrome virus (PRRSV) vaccination and infection was investigated. Forty-two weaned piglets were separated into seven groups and received three different diets: Low DON (1.09 ppm), High DON (2.81 ppm) or No DON. These three treatments were split further into either vaccinated (Ingelvac PRRSFLEX EU) and challenged with PRRSV 28 days post-vaccination, or only infected at day 28. A seventh group received no DON, no vaccination, and no infection. Two weeks after challenge infection, when pigs were euthanized, the number of IFN-γ producing lymphocytes in the blood of vaccinated animals was lower in pigs on High DON compared to animals on Low DON or No DON. Intracellular cytokine staining showed that vaccinated animals fed with the Low DON diet had higher frequencies of TNF-α/IFN-γ co-producing CD4^+^ T cells than the other two vaccinated groups, particularly in lung tissue. Vaccinated animals on High DON had similar viral loads in the lung as the non-vaccinated groups, but several animals of the Low DON or No DON group receiving vaccination had reduced titers. In these two groups, there was a negative correlation between lung virus titers and vaccine-specific TNF-α/IFN-γ co-producing CD4^+^ T cells located either in lung tissue or blood. These results indicate that after PRRSV vaccination and infection, high levels of DON negatively influence immune parameters and clearance of the virus, whereas low DON concentrations have immunomodulatory effects.

**Supplementary Information:**

The online version contains supplementary material available at 10.1007/s00204-023-03449-9.

## Introduction

Mycotoxins are secondary fungal metabolites which are produced by different fungal species, including *Fusarium, Aspergillus* and *Penicillium*. A global survey on 74,821 samples showed that mycotoxins contaminated 88% of tested feed and feed raw materials (e.g., maize, wheat, soybean) (Gruber-Dorninger et al. 2016). Among them, one of the major mycotoxins is the trichothecene type B deoxynivalenol (DON); it was found in 64% of the 74,821 samples consisting of cereals and finished feed (Gruber-Dorninger et al. 2019).

Farm animals can be seriously affected by DON as cereals of low quality are frequently dedicated to animal feed. Pigs are particularly sensitive to DON, due to their cereal-rich diet and their monogastric digestive system (Rotter et al. 1996). DON can induce anorexia, emesis, a decreased feed intake, weight loss, diarrhea and a higher sensitivity to infectious diseases due to its impact on the immune response (Pierron et al. 2016; Rotter et al. 1996). In a process designated ‘ribotoxic stress’, DON can bind to the 60S subunit of the ribosome, thereby affecting protein synthesis. This leads to an activation of various mitogen-activated protein kinases (MAPKs), ultimately resulting in oxidative stress, local inflammation and modulation of the immune response (Pestka 2010a, b; Garreau de Loubresse et al. 2014). Because of these toxic effects, a maximum dose of 0.9 mg/kg DON in pig feed is recommended by the European Feed Safety Authority (EFSA) (EFSA 2013).

The porcine reproductive and respiratory syndrome virus (PRRSV), which is grouped in the family *Arteriviridae*, induces substantial economic losses in the pig industry worldwide (Benfield et al. 1992; Renken et al. 2021). The respiratory form of PRRS leads to reduced animal welfare and reduced growth performance of pigs from all ages. The reproductive form of the disease induces late-term abortions, early farrowings, fetal death and the birth of weak piglets (Sinn et al. 2016a, b). Modified live virus vaccines (MLVs) have proven to be efficient in reducing the severity of clinical signs, as well as the duration of viremia and virus excretion (Scortti et al. 2006; Martelli et al. 2009). However, the co-exposure of pigs to PRRSV and DON may result in a modulation of the immune response to infection or vaccination, resulting in potential health impairment and economic losses (EFSA 2013).

So far, few in vitro and in vivo studies investigated the effects of DON on vaccine efficacy, comprising experimental vaccines against ovalbumin (OVA) (Lessard et al. 2015; Pinton et al. 2008) and tetanus toxoid (Overnes et al. 1997), against PRRSV in pigs (Rückner et al. 2022; Savard et al. 2015) and porcine parvovirus in mice (Choi et al. 2013). It has been noted that DON can cause either immunostimulatory or immunosuppressive effects (Payros et al. 2016). Hence, the aim of this study was to assess in more detail the effects of DON on the immune response following PRRSV vaccination and/ or infection. We hypothesized that DON would negatively affect the cellular and humoral immune response towards PRRSV vaccination, leading to reduced vaccine efficacy following a challenge infection.

We investigated the influence of DON (1.09 and 2.81 ppm) on PRRSV vaccination and subsequent challenge infection. These middle and low doses of DON were applied since constant feed surveillance minimizes the risk of high DON contamination in developed countries (Gruber-Dorninger et al. 2019). Average daily feed intake (ADI), average daily gain (ADG), and pathohistological changes in the jejunum were recorded. To monitor vaccine efficacy, histopathology of lung tissue and PRRSV viral loads in lung tissue, lymph nodes and serum were investigated. In addition, the local cellular immune response in lung tissue and lung draining lymph nodes was analyzed after necropsy of the animals two weeks post-challenge infection.

## Materials and methods (detailed description in Supplemental Materials and Methods)

### Study design

Forty-two crossbred three-week-old piglets (Large White x Pietrain) were randomly allocated to seven treatment groups; the groups were split among three different feeding regimens (ad libitum*,* starting from D-7) for 42 days: control feed (No DON: 0.05 ppm ± 0.08, *n* = 18), Low DON (envisaged: 0.9 ppm; after DON analysis: 1.09 ppm ± 0.03, *n* = 12) or High DON (envisaged: 3 ppm; after DON analysis: 2.81 ppm ± 0.44, *n* = 12), with two groups of pigs for each dose (Supplementary Figs. 1 and 2a, Supplementary Table 1). Seven days later (D0) three of the seven animal groups were immunized intramuscularly with a modified PRRSV live vaccine (Ingelvac PRRSFLEX^®^ EU, Boehringer Ingelheim Vetmedica, Ingelheim am Rhein, Germany) according to manufacturer’s instructions. Twenty-eight days post-vaccination the pigs in six of the seven groups were intranasally infected with a PRRSV-1 subtype 1 field isolate (AUT15-33, Gen Bank accession number MT000052.1). Pigs were euthanized over six days, from D41 to D46 post-vaccination (for logistical reasons), designated D44 ± 2 throughout the figures and text. Throughout the experiment, pigs were housed in a BSL2 animal care facility of the Vetmeduni Vienna, except the group with non-vaccinated non-infected DON-free fed animals (hereafter referred to as “Control”), which was housed in a regular pig facility. Sera were obtained for the detection of PRRSV-specific antibodies (D0, D14, D28, D31, D36, D44 ± 2) and for the determination of PRRSV viremia (D31, D36, D44 ± 2). Whole blood samples were collected to isolate peripheral blood mononuclear cells (PBMCs) at D0, D14, D28, and at the days of euthanasia (D44 ± 2). Additional information on feed and clinical monitoring is provided in the Supplemental Materials & Methods description.

### Isolation of PBMCs and lymphocytes

PBMCs were isolated from blood by gradient centrifugation. Lymphocytes were isolated from tracheobronchial lymph nodes (TBLNs) and lung tissue. Details are provided in Supplemental Material and Methods.

### Pathological examination

At necropsy days (D44 ± 2), a general pathological examination of the carcass was performed, with focus on the respiratory and intestinal tract. Details are given in Supplemental Materials and Methods.

### ELISpot assay to quantify PRRSV-specific IFN-γ-secreting cells

IFN-γ ELISpot assays were performed as previously described (Talker et al. 2015). Further details are given in Supplemental Materials and Methods.

### PRRSV-specific antibody production analyzed by PRRS X3 ELISA (IDEXX)

Sera were analyzed for PRRSV-specific antibodies using a commercial ELISA kit (IDEXX PRRS X3 Ab Test^®^, IDEXX Europe B.V., Hoofddorp, Netherlands). According to the manufacturer’s instructions, results were expressed as sample to positive control (S/P) optical density ratios with S/P ≥ 0.4 considered as positive.

### PRRSV quantification (viral load/challenge strain) in sera and tissues by quantitative reverse transcription-PCR (qRT-PCR)

Viral load was quantified in sera (target RNA copies/µL) at D31, D36 and D44 ± 2, corresponding to 3, 8 and 16 ± 2 days post-infection, respectively. In addition, in lung, tonsil, and TBLNs target RNA copies per mg tissue were analyzed after euthanasia (D44 ± 2). Viral RNA was extracted and qRT-PCR was performed as previously described (Kreutzmann et al. 2022). Further details are given in Supplemental Materials and Methods.

### Intracellular cytokine staining (ICS) and flow cytometry analysis

Freshly isolated PBMCs as well as lymphocytes from lung and TBLNs were subjected to ICS. Details are given in Supplemental Materials and Methods, Supplementary Table 2 and Supplementary Fig. 3.

### Statistical analysis

Data are presented as mean ± standard error of the mean (SEM) and analyzed using a normality test and analysis of variance (One way ANOVA) followed by Bonferroni’s multiple comparisons test; a *p* value < 0.05 was considered significant. Frequencies of TNF-α^+^IFN-γ^+^ CD4^+^ T cells in PBMCs or lung were correlated with the PRRSV load in the lung using the Pearson r correlation test. Statistical analyses were performed with GraphPad Prism 5 (GraphPad Software, San Diego, CA, USA).

## Results

### Influence of DON on growth performance and gut morphology

**Fig. 1:**
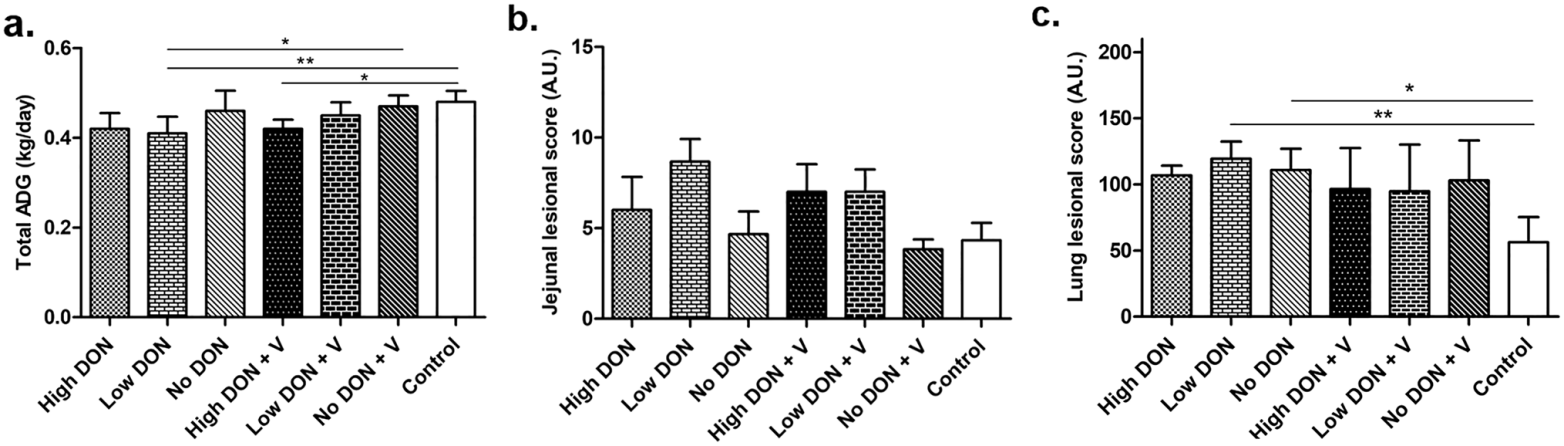
Influence of DON and PRRSV vaccination/infection on growth performance, gut pathology and lung pathology. For each graph, *n* = 6, *p < 0.05, **p < 0.01, *One way ANOVA,* Bonferroni’s multiple comparison test. **a** Total average daily gain (ADG) of pigs over the entire study period (D-7 to D44 ± 2). **b** Jejunal lesion score was assessed for each pig (lymphatic vessel dilation, cell vacuolation, cubic enterocytes, villus flattening, villus fusion, interstitial oedema, villi apical necrosis), considering the number of lesions and the severity of each lesion. **c** Lung lesion score was assessed for each pig (septal infiltration with mononuclear cells, pneumocytic hypertrophy and hyperplasia, intra-alveolar accumulation of inflammatory cells, perivascular accumulation of inflammatory cells and necrotic debris), taking into account the severity and extension of each lesion

ADI (Supplementary Fig. 2b) and ADG (Fig. 1a) were calculated for each group for the entire period of the trial. The estimated ADI per pig was lower for the High DON treated groups and higher for the animals in the control groups (Supplementary Fig. 2b). A slight decrease of the ADG was observed for the DON groups in comparison to the No DON groups, with or without vaccination (Fig. 1a). Significant reductions in ADG were found for the High DON + V group in comparison to the Control group, and for the Low DON group (without vaccination) in comparison to the No DON + V group and the Control group. Fecal scores, shown in Supplementary Fig. 2c, were slightly increased in the High DON groups between D5 and D22.

To assess the impact of DON on the intestine, microscopic jejunal lesions were evaluated (Fig. 1b). Overall, normal size of enterocytes, dilation of lymphatic vessels and cell vacuolation were found to different extents for all animals across the seven groups. Nevertheless, higher lesion scores (albeit not significant) were observed in animal groups fed with DON, compared to No DON and Control groups, mainly due to increased villi fusion and villi shortness (data not shown).

In summary, DON-feeding regimens caused reduced ADI and ADG and increased jejunal lesion scores. However, the effect of DON on these parameters was low and did not always reach significance.

### Pathology and viral loads after PRRSV infection

To monitor the effect of the PRRSV infection, rectal temperatures, viral loads in blood and organs (tonsil, TBLN and lung) as well as lung pathology were assessed. In all groups that underwent a PRRSV infection a slight increase of the body temperature above 40 °C was observed for up to 14 days after the challenge infection (D28) (Supplementary Fig. 4). These increases did not reach significance. Of note, no such increase was observed in the Control group.

After necropsy (D44 ± 2), macroscopic and microscopic lung lesions were evaluated. The gross lesions, tissue consistency and tan mottled areas of the lungs showed no significant differences between vaccinated and non-vaccinated groups but animals from the Control group had the lowest affected areas (data not shown). Histological scoring showed that the vaccinated groups tendentially had less lesions (but not significantly) than the non-vaccinated groups. There was no obvious influence of DON on the lung lesion score (Fig. 1c). Only the Low DON and the No DON groups had a significantly higher lesion score in comparison to the Control group.

**Fig. 2:**
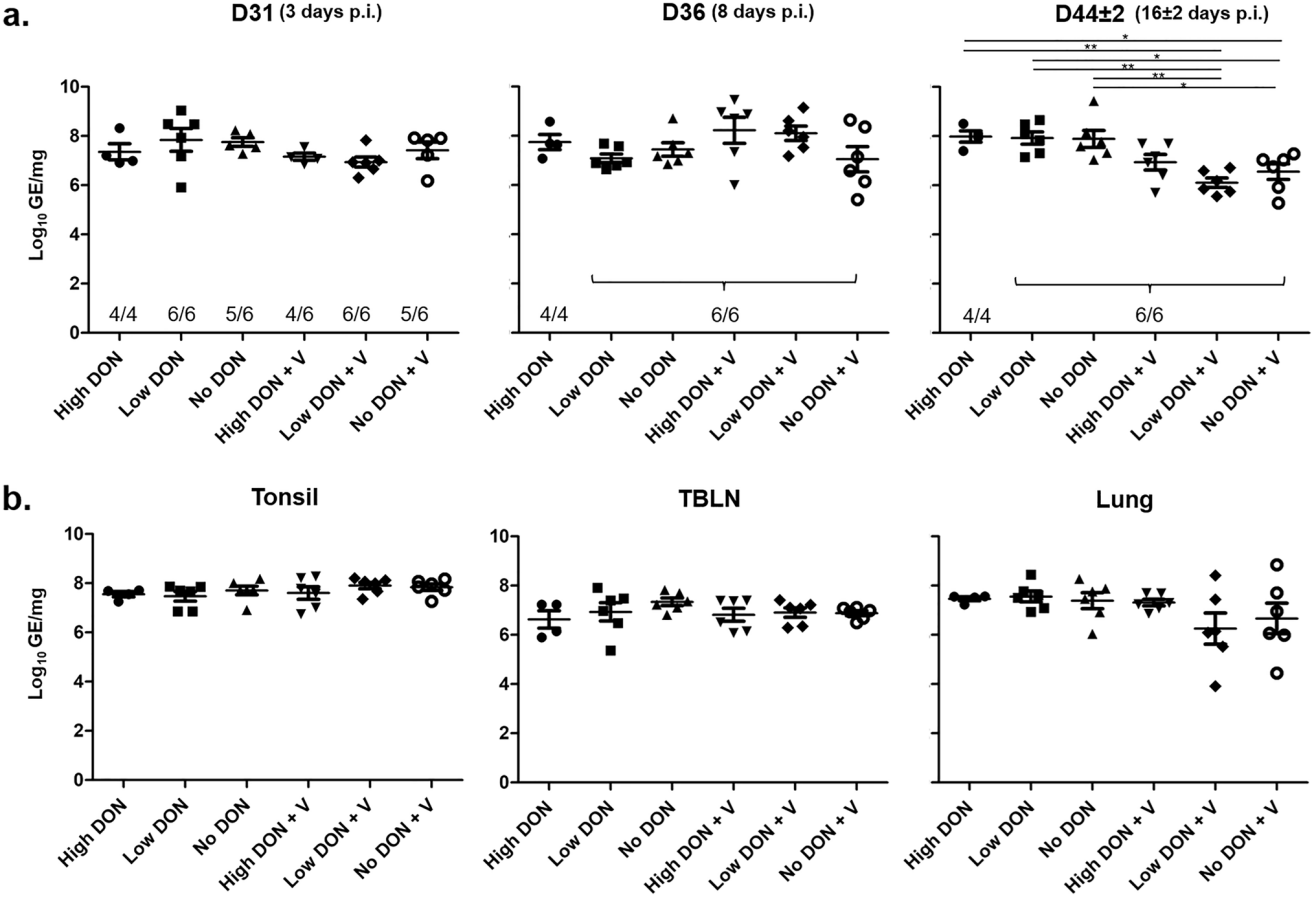
Effect of DON on PRRSV loads following vaccination and challenge infection. **a** PRRSV viremia measured in serum for all treatments at different times post-infection, D31, D36, D44 ± 2. **b** PRRSV loads measured at 16 ± 2 days p.i. (D44 ± 2) in different organs (tonsil, tracheobronchial lymph nodes [TBLNs] and lung) for all treatments. Numbers in (**a**) indicate the numbers of animals showing virus loads above the limit of detection. *N* = 4, 5 or 6, *p < 0.05, **p < 0.01, *One way ANOVA,* Bonferroni’s multiple comparison test

For groups that were infected with PRRSV, viral loads in the sera were investigated (Fig. 2a) at three (D31), eight (D36) and 16 ± 2 days (D44 ± 2, necropsies) p.i. Three days p.i. nearly all animals had become viremic, regardless of vaccination. Only one animal in the No DON, one animal in the No DON + V and two in the High DON + V groups stayed below the limit of detection. Eight days p.i. (D36), all animals in all groups had become viremic. Sixteen days p.i. (D44 ± 2) the viral load of the Low DON + V and the No DON + V groups were significantly lower than those of the non-vaccinated groups. In the High DON + V group viremia was also reduced in comparison to the non-vaccinated groups, but these differences did not reach significance. At necropsy (D44 ± 2) viral loads were also determined in tonsils, TBLNs and lung tissue (Fig. 2b). For tonsils and TBLNs, no obvious differences in viral loads between the six different groups were noted. This was similar in lung tissue, but some animals in the Low DON + V and the No DON + V had lower viral loads, resulting in a substantial heterogeneity in viral loads in these groups. But on the group level, no significant differences were found. Since sampling during necropsies was spread over six days, we also controlled if the sampling day had influence on obtained viral loads. No such influence was found; the viral load at necropsies did not correlate with the day of necropsy (Supplementary Fig. 5).

In summary, PRRSV infection led to viremia in all infected pigs, but this viremia was significantly reduced 16 ± 2 days p.i. in the Low DON + V and No DON + V groups. This coincided with a reduced viral load in the lung tissue of some animals within these two groups.

### PRRSV-specific IFN-γ producing lymphocytes and antibody response in the time course

**Fig. 3:**
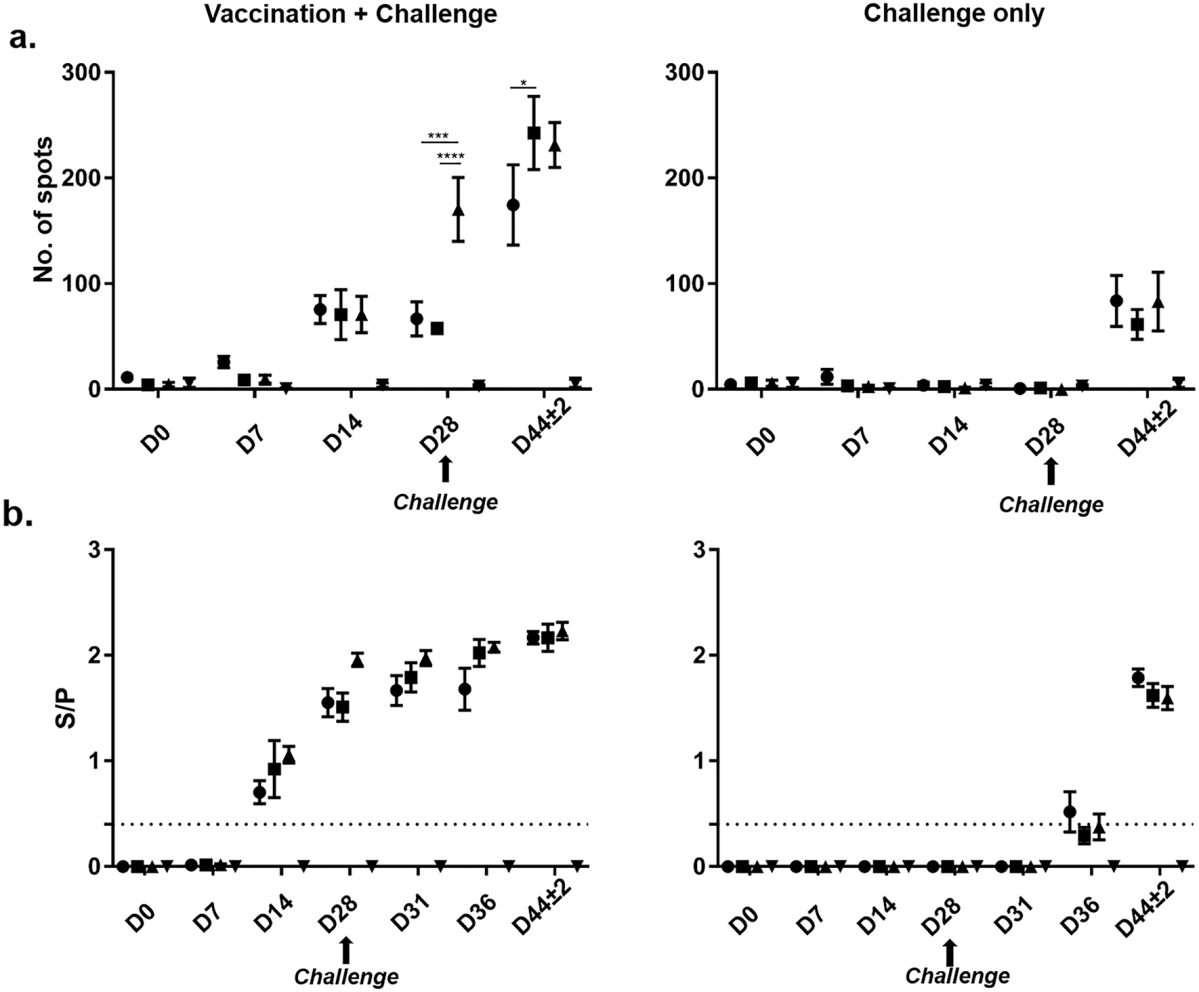
Influence of DON and PRRSV vaccination/infection on PRRSV-specific IFN-γ producing lymphocytes and antibodies. (●High DON, ■Low DON, ▲No DON, ▼Control). Graphs on the left represent groups vaccinated and challenged, on the right the groups challenged only. D0 is defined as the day of the vaccination with the modified PRRSV live virus vaccine (only vaccinated groups). **a** PRRSV vaccine-specific IFN-γ production analysed by ELISpot. IFN-γ producing cells (“spots”) per 3 × 10^5^ PBMC are displayed. **b** PRRSV-specific antibody production analysed by IDEXX PRRS X3 Ab Test^®^, expressed as S/P ratios. The ELISA was considered positive at an S/P ratio of ≥ 0.4 (dotted line). *p < 0.05, **p < 0.01, ***p < 0.001, ****p < 0.0001, *One way ANOVA,* Bonferroni’s multiple comparison test

The PRRSV antigen-specific IFN-γ production of blood-derived lymphocytes was analyzed by ELISpot over the time course of the study (Fig. 3a). PRRSV vaccination induced IFN-γ producing lymphocytes in the blood of all vaccinated animals, regardless of the diet from D14 onwards (Fig. 3a, left panel). At D28 post-vaccination, DON groups had significantly reduced numbers of IFN-γ producing lymphocytes compared to the No DON + V group. At the day of necropsy (D44 ± 2), the High DON + V group had still significantly lower numbers of IFN-γ producing cells in the blood in comparison to the Low DON + V group, which now had reached similar numbers as the No DON + V group. For non-vaccinated, challenged pigs, a low number of IFN-γ producing lymphocytes was detectable at D44 ± 2 (16 ± 2 days p.i.), with no marked differences between groups (Fig. 3a, right panel). IFN-γ spots of the Control group stayed at baseline levels on all investigated time points.

The PRRSV-specific IgG production in serum was evaluated by a commercially available ELISA (Fig. 3b). This ELISA is not designed for quantitative analysis; hence we did not perform statistical analyses. However, we observed that the PRRSV vaccination induced the production of PRRSV-specific antibodies two weeks (D14) post-vaccination (Fig. 3b, left panel). At D28 post-vaccination DON groups showed reduced S/P ratios of vaccine specific antibodies in the blood compared to the No DON + V group, coinciding with results of IFN-γ producing lymphocytes (Fig. 3a). At D36 post-vaccination, corresponding to 8 days p.i., the No DON + V group showed higher S/P ratios than the High DON + V group while the Low DON + V group had similar S/P values as the No DON + V group. Finally, at D44 ± 2, S/P ratios in all three vaccinated groups reached similar levels, regardless of DON feeding. In non-vaccinated groups, S/P ratios were very similar at D44 ± 2, regardless of DON-feeding.

In summary, IFN-γ producing lymphocytes and antibodies in blood were affected by the high and the low dose of DON at D28 (i.e. four weeks post-vaccination). However, post-challenge, animals receiving the low dose of DON quickly reached the levels of the No DON group for both parameters.

### Local cytokine production of CD4^+^ T cells after PRRSV vaccination and challenge infection

**Fig. 4:**
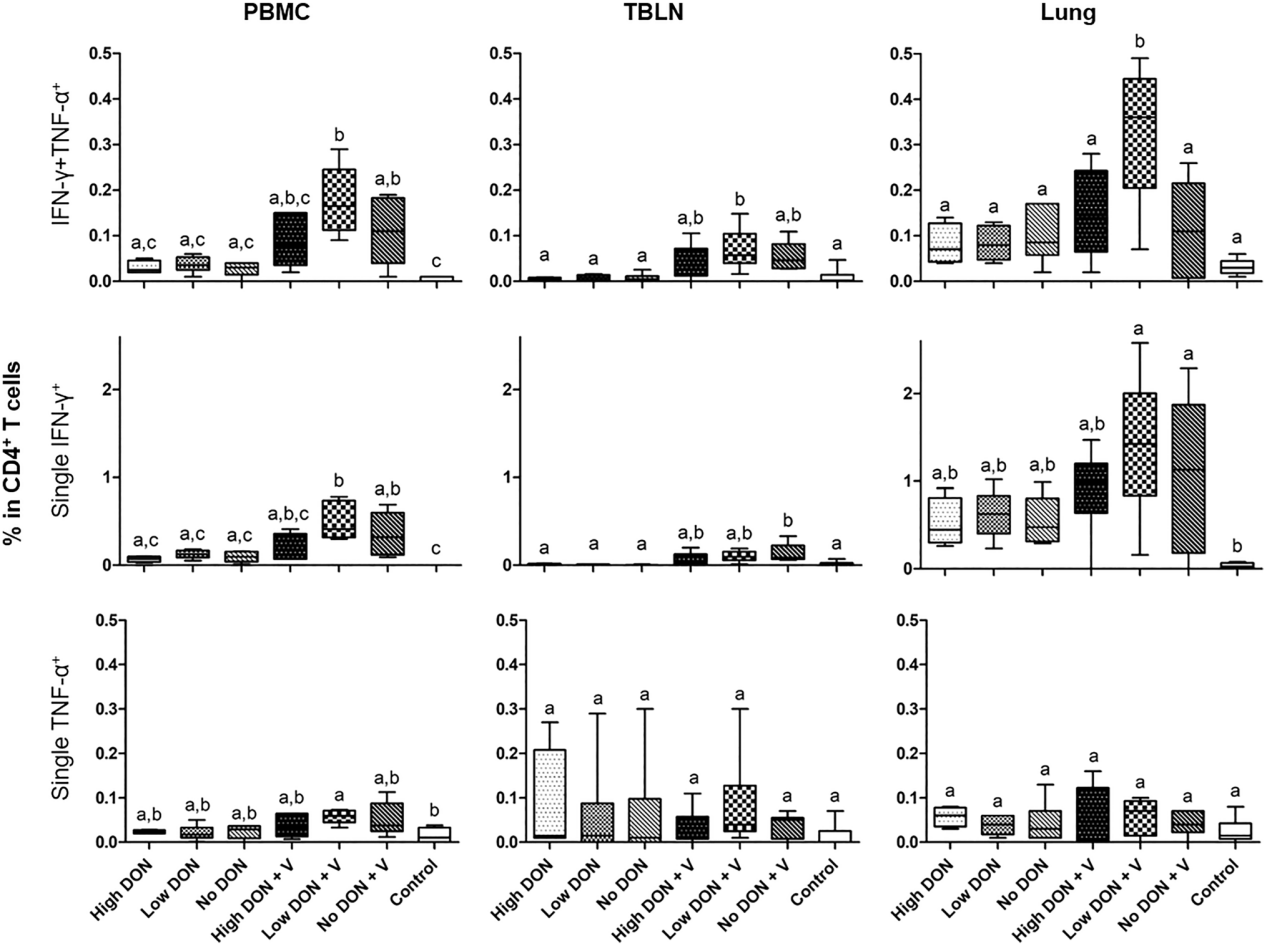
Influence of DON and PRRSV vaccination/infection on cytokine production in CD4^+^ T cells. Graphs show the percentages of cytokine producing CD4^+^ T cells within total CD4^+^ T cells, isolated from blood (PBMC, left), tracheobronchial lymph nodes (TBLNs, middle) and lung tissue (right) at D44 ± 2. Top: percentage of IFN-γ^+^TNF-α^+^ cells**,** center: single IFN-γ^+^ cells, bottom: single TNF-α^+^ cells (gating strategy shown in Supplementary Fig. 3). *n* = 6, *p* < 0.05, *One way ANOVA,* Bonferroni’s multiple comparison test. Samples with different letters differ significantly

To analyze the T-cell response also locally, the frequencies of PRRSV-specific single TNF-α^+^, single IFN-γ^+^ and TNF-α^+^IFN-γ^+^ producing CD4^+^ T cells isolated from blood, TBLNs and lung tissue were investigated by flow cytometry at D44 ± 2 (16 ± 2 days p.i.) (Fig. 4).

We observed a significant increase of the frequency of TNF-α/IFN-γ co-producing CD4^+^ T cells in all organs (blood, TBLN and lung) for the Low DON + V group in comparison to the non-vaccinated groups and the Control group, as well as in comparison to all other groups in the lung (Fig. 4, top row). For the single IFN-γ producing CD4^+^ T cells (Fig. 4, middle row), the Low DON + V group in blood showed an increase of the frequency in comparison to the Control group and the non-vaccinated groups; there was also a significant difference between the No DON + V and the Control group. In TBLN, only the No DON + V group had a significantly increased frequency of single IFN-γ^+^ CD4^+^ T cells in comparison to the non-vaccinated groups and the Control group. In the lungs, the Low DON + V and the No DON + V groups showed a significant increase of single IFN-γ^+^ CD4^+^ T cells in comparison to the Control group. For single TNF-α^+^ producing CD4^+^ T cells, a significant increase of the frequency in the blood was only observed for the Low DON + V group in comparison to the Control group (Fig. 4, bottom row).

For TNF-α/IFN-γ co-producing CD4^+^ T cells in the blood, these data confirm data from the IFN-γ ELISpot at D44 ± 2. However, in the ICS experiments even higher frequencies of TNF-α/IFN-γ co-producing CD4^+^ T cells were found in the Low DON + V group compared to the No DON + V group.

### Correlation of the lung viral load and the frequency of TNF-α/IFN-γ co-producing CD4^+^ T cells

**Fig. 5:**
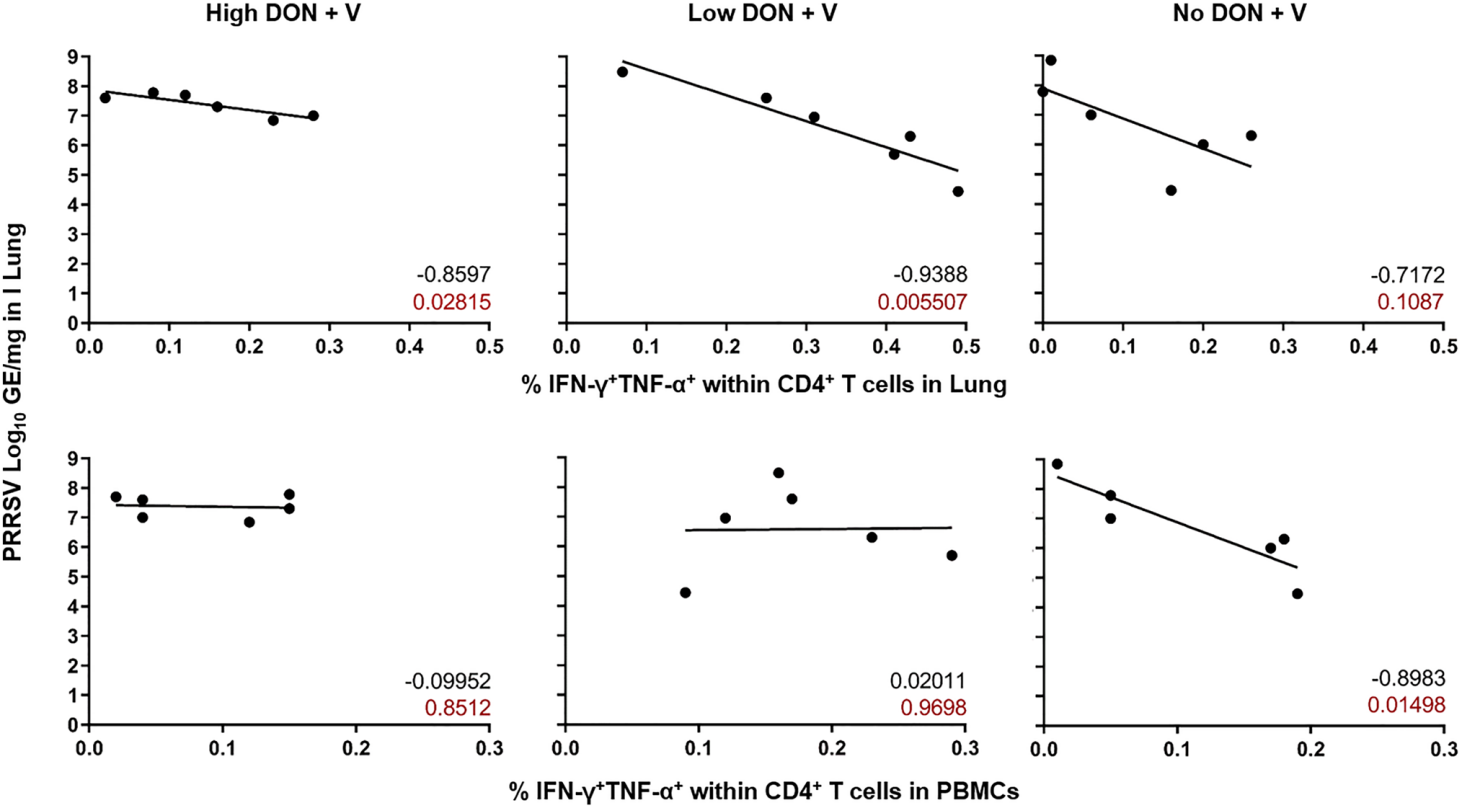
Correlation of lung viral loads with the frequencies of TNF-α/IFN-γ co-producing CD4^+^ T cells. Graphs display percentages of TNF-α/IFN-γ co-producing CD4^+^ T cells on the x-axis and PRRSV viral loads on the *y*-axis at D44 ± 2. Top: TNF-α/IFN-γ co-producing CD4^+^ T cells isolated from lung tissue, bottom: TNF-α/IFN-γ co-producing CD4^+^ T cells isolated from blood (PBMC). Coefficient of correlation and *p* value are indicated for each graph in black and in red, respectively. *n* = 6, *p* < 0.05, Correlation test of Pearson r

Our analyses of viral loads in lung tissue showed a notable variation among individual animals of the Low DON + V and the No DON + V groups (Fig. 2b, right panel). We hypothesized that these individual differences may have been reflected in the TNF-α/IFN-γ T-cell response, which, in the past, had been shown to be associated with vaccine-induced protection in pigs and humans (Franzoni et al. 2013; Seder et al. 2008). Hence, we tested whether there was a correlation of the viral load in lung tissue with the frequency of TNF-α^+^IFN-γ^+^ CD4^+^ T cells in the lungs (Fig. 5, top panels) and blood (Fig. 5, bottom panels). We found a significant negative correlation for the High DON + V and the Low DON + V group between lung viral loads and the frequency of TNF-α^+^IFN-γ^+^ CD4^+^ T cells. This negative correlation was also found for the No DON + V group but did not reach significance. Concerning TNF-α^+^IFN-γ^+^ CD4^+^ T cells in the blood, only the No DON + V group showed a significant negative correlation of the viral load in lung.

## Discussion

Effects of DON on the immune response and inflammation have been described across multiple species (Pestka 2010a; Payros et al. 2016; Pierron, et al. 2016). Young pigs are particularly sensitive to this mycotoxin, causing anorexia, vomiting and growth impairment even at low doses in feed (Holanda and Kim 2021). In addition, there are suppressing effects on the immune system at high doses or stimulating effects at low doses (Pierron et al. 2016). An EFSA evaluation concluded that there is a wide range of doses at which effects of DON are pertinent, from 0.7 to 8 ppm of DON for NOAELs (Non Observed Adverse Effect Level) and from 0.35 to 13 ppm of DON for LOAELs (Lowest Observed Adverse Effect Level) (Knutsen et al. 2017).

However, the influence of DON on vaccine-specific immune responses and consecutive challenge infections has not been investigated in much detail. This also applies to PRRS, which is one of the economically most relevant diseases affecting pigs (Lunney et al. 2016). In weaned and growing pigs PRRS leads to respiratory signs and reduced growth performance (Nathues et al. 2017). In this study, we aimed to investigate the humoral and the T-cell response after PRRSV vaccination and following challenge infection under the influence of two doses of DON which are relevant under field conditions. The targeted dose of 0.9 ppm (final concentration obtained 1.09 ppm) mimics a contamination level to which piglets are regularly exposed on farms (Knutsen et al. 2017). The high dose of 2.81 ppm of DON (initially envisaged 3.0 ppm) corresponds to a dose of intermediate toxicity that is also frequently found in feed used on pig farms (Knutsen et al. 2017).

For our study, we used a licensed PRRSV-1 MLV for vaccination and challenged with the heterologous PRRSV-1 strain AUT15-33. This PRRSV isolate has been shown to be highly virulent in experimental infection studies with growing pigs and pregnant sows (Kreutzmann et al. 2021, 2022; Dürlinger et al. 2022). In our study, we observed viral loads in the serum of around 8 log_10_ genome copies/mL (Fig. 2a), which is in accordance with two previous studies using this isolate (Kreutzmann et al. 2022; Dürlinger et al. 2022). Regarding lung pathology and body temperature, we did not observe clear differences between vaccinated and infected and only infected pigs, irrespective of the DON feeding regimen. This was different in the studies by Kreutzmann et al. (2021) and Dürlinger et al (2022), where significant reductions in lung pathology were found in vaccinated animals compared to non-vaccinated animals. These differences may be due to a different age of pigs used in the study by Kreutzmann et al. (2021; vaccination at the first day of life, challenge infection at day 28) or much larger animal groups and different housing facilities in the study by Dürlinger et al. (2022).

Two previous publications reported on the influence of DON either after PRRSV vaccination or after PRRSV infection, but not with both conditions together (Savard et al. 2014, 2015). Savard et al. (2014) investigated the influence of 2.5 and 3.5 mg/kg DON in feed on PRRSV infection. Piglets fed 3.5 mg/kg DON had higher lung lesion scores 21 days p.i., albeit viral load in lung tissue was in tendency reduced in DON-fed pigs. PRRSV-specific antibody S/P ratios were slightly reduced compared to DON-free fed pigs. Of note, Sarvard et al. (2014) used a PRRSV-2 strain for infection, with a history of clinical signs in the field. Sarvard et al. (2015) reported on the influence of DON contaminated feed on PRRSV vaccination. Viremia of the vaccine virus was reduced in pigs on 3.5 mg/kg DON. PRRSV antibody S/P ratios were also reduced at this dose between 13 and 34 days post-vaccination.

A more recent study by Rückner et al. (2022) investigated the effect of 1 and 2 mg/kg DON in feed on PRRSV vaccination and challenge infection in the same animals. Different to our study, a PRRSV-2 MLV vaccine was used. These authors found reduced PRRSV antibody S/P ratios for some (but not all) animals on 2 mg/kg DON feed compared to DON free control pigs two weeks post-vaccination. This animal-to-animal variation of antibody S/P ratios was also observed following challenge infection with a heterologous PRRSV-1 strain (applied two weeks post-vaccination) for the 2 mg/kg DON-fed pigs, at one- and two-weeks post-challenge. At the same time, viral loads in lung, conjunctiva and liver, analyzed 14 days post-challenge, did not differ between DON-fed and DON-free pigs.

In our study we observed a reduction in PRRSV ELISA S/P ratios 28 days post-vaccination, which coincided with a reduced number of IFN-γ producing lymphocytes (Fig. 3b and a, respectively). Hence, one common finding of the three studies addressing PRRSV-specific antibodies following vaccination (Sarvard et al. 2015; Rückner et al. 2022; this study) is a reduction in antibody S/P ratios at DON concentrations of 2 ppm or higher. The time point of this effect post-vaccination seems to vary (Sarvard et al. 2015 and Rückner et al. 2022: day 13/14 onwards, our study: day 28) but may also be influenced by the type of PRRSV vaccine. Sarvard et al. (2015) and Rückner et al. (2022) used a PRRSV-2 MLV, whereas in our study a PRRSV-1 MLV was used. Following challenge infection, in our study and in Rückner et al (2022) reduced antibody S/P ratios persisted 7–14 days post-challenge infection onwards with DON doses of 2.81 ppm or 2 mg/kg, respectively, in comparison to pigs on DON free diets. Of note, in our study these reduced S/P antibody ratios at 2.81 ppm appeared to be transient, since no difference between the vaccinated and challenged groups was found at necropsy (Fig. 3b, left panel).

Different to the previous studies addressing PRRSV vaccination/ infection and DON, we also analyzed T-cell responses by IFN-γ ELISpots and ICS for TNF-α and IFN-γ. We focused on those cytokines since previous work on PRRSV vaccination suggested that IFN-γ producing lymphocytes may serve as a correlate of protection following PRRSV vaccination (Martelli et al. 2009; Kreutzmann et al. 2021; Ferrarini et al. 2015; Madapong et al. 2020). In accordance with this, our findings show that higher frequencies of TNF-α/IFN-γ co-producing CD4^+^ T cells in the blood of pigs on a DON-free diet show a negative correlation with PRRSV viral loads in the lung (Fig. 5, lower right). One interesting result of our study was a significantly increased frequency of TNF-α^+^IFN-γ^+^ producing CD4^+^ T cells in lung tissue of pigs in the Low DON + V group, compared to all other groups (Fig. 4, top right panel). This finding coincided with a negative correlation of these cells with PRRSV loads in the lung (Fig. 5, top middle panel).

Immunostimulatory effects of low doses of DON on T cells have been described before. In vitro work from the authors of this study could show that mitogen-induced proliferation and the expression of the co-stimulatory molecules CD27 and CD28 of porcine T cells is negatively affected by doses ≥ 0.8 μM of DON (Vatzia et al. 2019). However, a follow-up study revealed that a dose of 0.8 μM of DON is capable to increase T-bet expression and production of IFN-γ and TNF-α in porcine CD4^+^ and CD8^+^ T cells (Vatzia et al. 2020), reminiscent of in vivo observations in this study. However, such effects seem to occur in a small dose-dependent window; in vitro work by Hlavová et al. (2020) showed that porcine CD4^+^ and γδ T cells have a reduced production of IFN-γ, TNF-α and IL-17 following polyclonal stimulation in the presence of 0.034 µM DON.

The underlying mechanisms for these phenomena remain somewhat elusive. DON binds to the A-site of the 60S subunit of ribosomes (Garreau de Loubresse et al. 2014) and induces mitogen-activated protein kinases (MAPKs) like Erk1/2 and p38 (Moon 2002; Shifrin and Anderson 1999). In activated T cells, these MAPKs drive calcium influx and the expression of transcription factors like NFAT and AP-1 (Farber 2009), thereby supporting IL-2 production and ultimately T cell proliferation. The pigs in this study received an intranasal inoculation and it has been shown that PRRSV drives the production of type-1 polarizing cytokines (IL-12, IFN-γ) on the transcriptional and protein level in the lung (Sánchez-Carvajal et al. 2022; Nazki et al. 2020). Hence it is conceivable that this local cytokine milieu together with the proliferation-supporting effects of DON drove the expansion of TNF-α/IFN-γ producing, local memory CD4 T cells, initially induced by the vaccination. This effect may have even compensated for the suboptimal induction of IFN-γ producing lymphocytes (which in their majority most probably consisted of CD4 T cells) observed in the Low DON + V group 28 days post-vaccination (Fig. 3a, left panel).

In summary, this study provides further evidence that under particular circumstances DON may have immunostimulatory properties, even in an in vivo setting. However, we also provide evidence that in the context of PRRSV vaccination, DON concentrations above the EFSA recommendation of 0.9 mg/kg feed, regularly found in the field, are detrimental for the vaccine-induced antibody production and negatively affect accompanying PRRSV-specific T cell responses.

## Supplementary Information

Below is the link to the electronic supplementary material.

Supplementary file1 (DOCX 1771 KB)

## Data Availability

The datasets generated during and/or analysed during the current study are available from the corresponding author on reasonable request (if not contained in the Supplemental Material).
