# Supplementary material for: Influence of deoxynivalenol-contaminated feed on the immune response of pigs after PRRSV vaccination and infection
Source: Arch Toxicol. 2023 Feb 13;97(4):1079–89. doi: 10.1007/s00204-023-03449-9 (PMC10025202; doi:10.1007/s00204-023-03449-9)
Supplement: Supplementary file 1 — Supplementary file1 (DOCX 1771 KB) [file 204_2023_3449_MOESM1_ESM.docx]

**Additional information**

**Influence of deoxynivalenol-contaminated feed on the immune response of pigs after PRRSV vaccination and infection**

Alix Pierron^1a*^, Eleni Vatzia^1b*^, Maria Stadler^1^, Kerstin H. Mair^1^, Selma Schmidt^1b^, Melissa R. Stas^2^, Sophie Dürlinger^2^, Heinrich Kreutzmann^2^, Christian Knecht^2^, Gyula Balka^3^, Julia Lagler^1c^, Marianne Zaruba^4^, Till Rümenapf^4^, Armin Saalmüller^1^, Elisabeth Mayer^5^, Andrea Ladinig^2^, Wilhelm Gerner^1b^

*^1^ Institute of Immunology, Department of Pathobiology, University of Veterinary Medicine, Vienna, Austria*

*^2^ University Clinic for Swine, Department for Farm Animals and Veterinary Public Health, University of Veterinary Medicine, Vienna, Austria*

*^3^ Department of Pathology, University of Veterinary Medicine, Budapest, Hungary*

*^4^ Institute of Virology, Department of Pathobiology, University of Veterinary Medicine, Vienna, Austria*

*^5^ DSM - BIOMIN Research Center, Tulln, Austria*

*^a^ Present address:* *ENVT (National Veterinary School of Toulouse), IHAP, Toulouse, France*

*^b^ Present address: The Pirbright Institute, Woking, United Kingdom*

*^c^ Present address: Institute of Virology, University of Zurich, Switzerland*

*^*^ Equal contributors*

**Corresponding author**

Wilhelm Gerner; E-Mail: wilhelm.gerner@vetmeduni.ac.at

**Supplementary Materials & Methods**

**Study design and clinical monitoring: additional information**

Piglets were obtained from a conventional farm free of PRRSV in Lower Austria. On arrival at day -14 (D-14), piglets were weighed and divided into seven groups of six animals according to their body weight (Online resource 2). Feed rations were provided by Biomin Holding GmbH, Tulln (Online resource 3). Each feed regimen consisted of two different mixtures (FAF1 and FAF2) which were adapted to the age of the piglets (Online resource 4a). For infection, a PRRSV-1, subtype 1 field isolate, designated as AUT15-33 (GenBank MT000052.1; Sinn et al. 2016) was used. One mL of virus suspension with 10^5^ TCID_50_ per mL was administered into each nostril. Euthanasia was done by intracardial administration of T61^®^ (T61^®^: embutramide, mebezonium iodide, and tetracaine hydrochloride, 1 mL/10 kg BW, Intervet GesmbH, Vienna, Austria) during anesthesia by prior intramuscular injection of ketamine (Narketan^®^ 100 mg/ml, Vetoquinol Österreich GmbH, Vienna, Austria; 10 mg/kg body weight) and azaperone (Stresnil^®^ 40 mg/ml, Elanco GmbH, Cuxhaven, Germany; 1.5 mg/kg body weight).

Vaccinated and non-vaccinated animals were kept in separate compartments of the BSL2 facility. Rectal temperature and clinical signs of the pigs were monitored daily, as described elsewhere (Stadler et al. 2018). Fever was defined as a body temperature higher than 40°C for three consecutive days. Blood samples were taken by puncture of the V. cava cranialis or V. jugularis as indicated in the timeline (Online resource 2). For calculation of the average daily weight gain, piglets were weighed every week. For calculation of the average daily intake (ADI) within each animal pen (n=6) feed added daily and remaining feed at the end of each week was weighed.

**Sample collection, isolation of PBMCs and lymphocytes**

Blood samples were collected in Lithium-Heparin tubes (Primavette^®^, KABE Labortechnik, Nümbrecht, Germany). PBMCs were isolated using lymphocyte separation medium of 1.077 g/mL density (Pancoll human, PAN Biotech, Aidenbach, Germany; 30 min at 920 x g). Freshly isolated PBMCs were counted by a cell counter (XP-300 Haematology Analyser, Sysmex Europe GmbH, Norderstedt, Germany). For serum collection, blood was centrifuged at 3000 rpm, for 10 min, and frozen at -20°C until further use.

Isolation of lymphocytes from tracheobronchial lymph nodes (TBLNs) and lung tissue was performed as described elsewhere (Reutner et al. 2012; Sassu et al. 2017). To standardize the isolation of lymphocytes from lung tissue, one piece of the middle part of the caudal left and right lobe were collected from each pig, independent of the presence of lesions.

### Pathological examination

TBLNs, lung (each lobe) and jejunum were collected and gross lesions were documented. For histopathology analyses, jejunal and lung samples were fixed in 10% neutral buffered formalin, embedded in paraffin wax, processed in 3-µm-thick paraffin-embedded sections and stained with hematoxylin and eosin. To assess histological changes in the jejunum, gut sections were assessed via light microscopy (NIKON 90i, NIS-Elements AR, following a previously described method (Bracarense et al. 2012)). The frequency and severity of each lesion (lymphatic vessel dilation, cell vacuolation, cubic enterocytes, villus flattening, villus fusion, interstitial oedema, villi apical necrosis) were considered in the score; according to their intensity or observed frequency, scores from 0 to 3 ('no lesion’ to ‘large extent’) were applied. For each lesion, the score of the extent was multiplied by the severity factor (from 1, ‘mild’ to 3, ‘severe’), leading to a maximum score of 38.

For the lungs, gross lesions, tissue consistency and tan mottled areas of the seven lobes (left cranial, left middle, left caudal, right cranial, right middle, right caudal and accessory) were evaluated and results were expressed using the American table of Straw et al. (1986). Slides for histopathology were scanned (Pannoramic Midi slide scanner, 3DHistech, Budapest, Hungary) and then examined via the SlideViewer software (3DHistech) for presence and quantity of the following parameters as described previously (Kreutzmann et al. 2021). In summary, five different lesions were scored for each of the seven lung lobes based on their severity and extension (from 0 to 3): septal infiltration with mononuclear cells, pneumocytic hypertrophy and hyperplasia, intra-alveolar accumulation of inflammatory cells, perivascular accumulation of inflammatory cells and necrotic debris. For each lesion, the individual scores were added, leading to a maximum lesion score of 42 and a maximum total score for all five lesions of 210 per animal (Balka et al. 2013).

**ELISpot assay to quantify PRRSV-specific IFN-γ-secreting cells**

ELISpot plates (MSIPS4510, Merck Millipore, Darmstadt, Germany) were coated overnight with mouse anti-pig IFN-γ monoclonal antibody (mAb) (clone pIFNγ-I, Mabtech, Nacka Strand, Sweden), at 10 μg/mL. After washing, freshly isolated PBMCs were added to wells (3 × 10^5^ PBMCs per well in triplicates) in RPMI 1640 with stable glutamine (PAN Biotech, Aidenbach, Germany) supplemented with 10% (v/v) inactivated fetal calf serum (FCS, Merck, Darmstadt, Germany), 100 IU/mL penicillin and 0.1 mg/mL streptomycin (PAN Biotech). Cells were stimulated either with medium only (negative control) or the Ingelvac PRRSFLEX^®^ EU vaccine, reconstituted in cell culture medium and adjusted to a multiplicity of infection (MOI) of 0.1, for 24h at 37°C. Then, plates were washed and incubated with biotin-labeled mouse anti-pig IFN-γ mAb (clone P2C11, Mabtech, 0.5 mg/mL) followed by incubation with streptavidin alkaline phosphatase (Roche, Mannheim, Germany). Finally, 5-bromo-4-chloro-3-indolyl phosphate/nitro blue tetrazolium substrate (Sigma-Aldrich, Vienna, Austria) was added for spot development. Spots were analyzed with an AID ELISpot reader (AID, Straßberg, Germany). Frequencies of IFN-γ secreting cells were calculated by subtracting spot numbers of medium-incubated cultures from spot numbers detected in cultures with PRRSV vaccine stimulation.

**PRRSV quantification (viral load of challenge strain) in sera and tissues by quantitative reverse transcription-PCR (qRT-PCR)**

Viral load was quantified in sera (target RNA copies/mL) at D31, D36 and D44 ± 2, corresponding to 3, 8 and 14 days post infection, respectively. In addition, in lung, tonsil, and TBLNs target RNA copies per mg tissue were analyzed after euthanasia (D44 ± 2). Viral RNA was extracted and qRT-PCR was performed as previously described (Kreutzmann et al. 2022).

Shortly, viral RNA was extracted with the cador Pathogen Kit for viral nucleic acid purification in a QiaCubeHT instrument (QIAGEN GmbH, Hilden, Germany) according to the manufacturer's protocol. Two microliters of the eluted RNA were used for ORF7-specific RT-qPCR using the Luna Onestep RT PCR Kit (New England Biolabs). The primer sequences were adapted from Egli et al. (2001) to fit the sequence of PRRSV-1 strain AUT15-33 (PRSq1 forward: TCAACTGTGCCAGTTGCTGG, PRSq2 reverse: TGRGGCTTCTCAGGCTTTTC, and PRSq3 probe: 5′Fam-CCCAGCGYCRRCARCCTAGGG Tamra-3′). For RT-qPCR of the vaccine strain contained in Ingelvac PRRSFLEX EU, the primer set PRSq1 forward: TCAACTGTGCCAGTTGCTGG, PRSq4 reverse: TGTGGCTTCTCAGGCTTCTTC, and PRSq5 probe: 5′Fam-CCCAGCGCCAGCAAYCTAGGG Tamra-3′ were employed.

The absolute quantity of the genome equivalents (GE) was calculated from serially diluted SP6 transcripts of cloned AUT15-33 cDNA fragment 13261−3′ end in a pGEM-T (Promega GmbH, Germany) plasmid (pLS69). Transcripts were generated with 0.5 μg of AclI linearized pLS69 plasmid DNA using 20 units of SP6 polymerase (New England Biolabs) in a 20-μl reaction. Template DNA was digested with DNAse I (New England Biolabs), and the RNA was purified using the RNeasy Kit (QIAGEN). The RNA concentration was determined with a Quantus fluorometer and an RNA-specific fluorescent dye (Promega). The number of genome molecules was calculated by multiplication of the RNA concentration with Avogadro's number divided by the molecular mass of an AUT15-33-specific SP6 transcript. Quantitative PCR was done in an Applied Biosystem 7300 instrument (Applied Biosystems, Thermo Fisher Scientific Inc., USA).

**Intracellular cytokine staining (ICS) and flow cytometry (FCM) analysis**

Freshly isolated cells were incubated overnight at 37°C in 96-well round-bottom plates at 5x10^5^ cells per well in a final volume of 200µL, with either cell culture medium (negative control) or reconstituted vaccine (MOI = 0.1). Then, cells were incubated 4 hours in the presence of Brefeldin A (1µg/mL, BD GolgiPlug™, BD Biosciences, San Jose, CA, USA).

Thereafter, the cells were harvested and labelled with the antibodies and reagents listed in Supplementary Table 2. A consecutive seven-colour FCM staining was performed; each antibody incubation step lasted for 20 min at 4 °C and was followed by two washing steps with PBS + 3% FCS. For discrimination of dead cells, the Live/Dead fixable viability dye eFluor780 (Thermo Fisher Scientific, Waltham, MA, USA) was used. After labelling of cell surface markers, cells were fixed and permeabilized for 20 min with BD Cytofix/Cytoperm and washed with BD Perm/Wash (BD Biosciences) according to manufacturer’s instructions. Finally, mAbs against IFN-γ and TNF-α were added.

After the last incubation, the cells were washed in BD Perm/Wash and resuspended in 50µL of BD Perm/Wash for overnight storage. The following day, 150µL of BD Perm/Wash was added, cells were transferred to 5 mL tubes and analyzed with a FACSCanto II flow cytometer (BD Biosciences). At least 1 × 10^6^ lymphocytes were recorded per sample. Obtained data were analyzed by FlowJo software version 10.5.3. (BD Biosciences). The FlowJo FlowAI algorithm was used for data cleaning. FlowAI is an algorithm that checks fluorescence and light scatter parameters over time and excludes events outside the statistical norm. The applied gating strategy together with representative raw data is shown in Supplementary Figure 3. ICS data was further processed by subtracting the percentage of cytokine producing cells in the medium condition from the percentage of cytokine producing cells restimulated with PRRSV vaccine antigen.

**
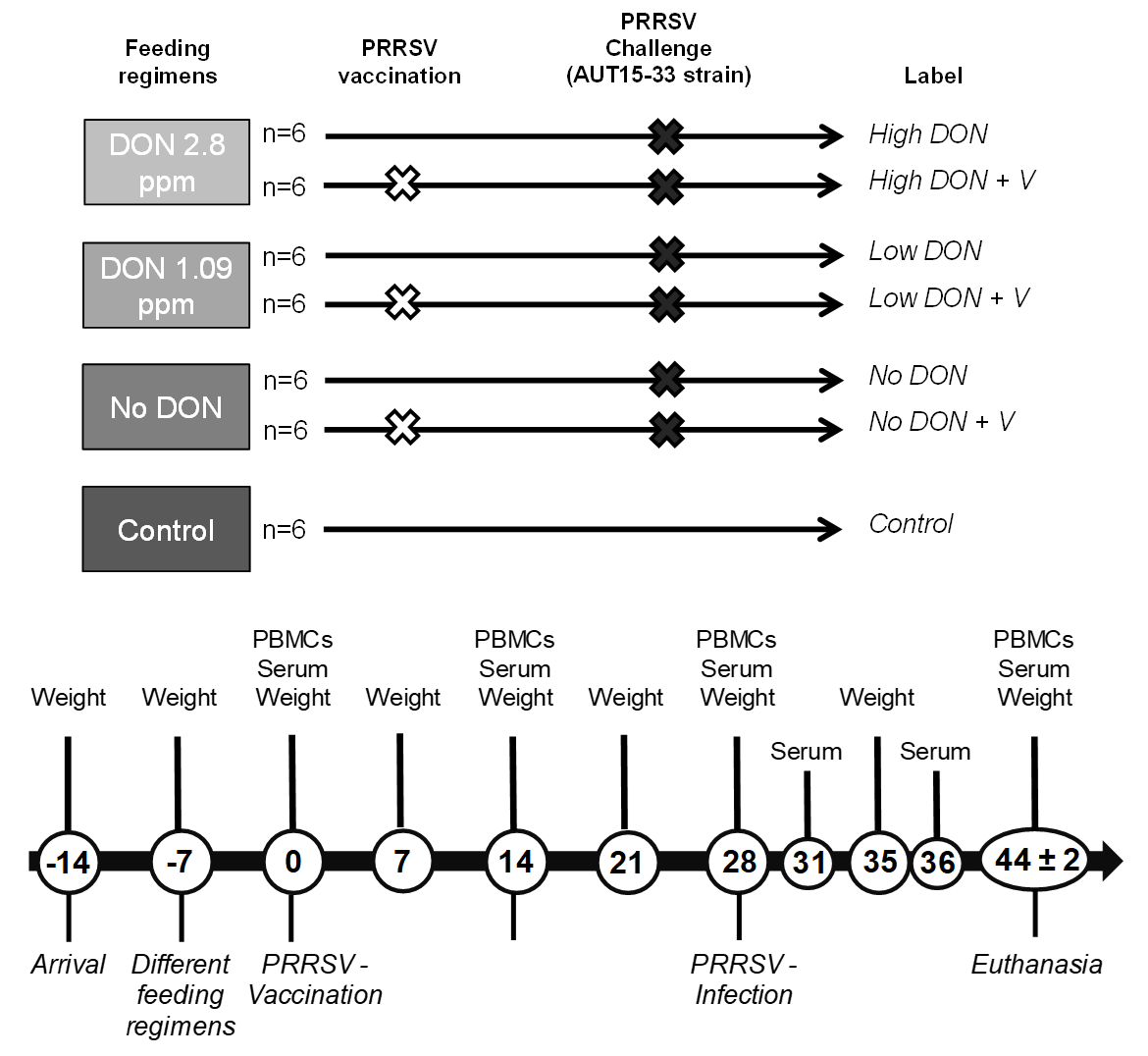
**

**Supplementary Figure 1:** Design of animal experiment. On arrival at day (D) -14, 42 pigs were weighed and allocated into 7 groups. Two groups received DON at 1.09 ppm (Low DON groups), 2 groups received DON at 2.8 ppm (High DON groups) and 2 groups had no DON in their diet (No DON groups). Six groups were infected with PRRSV at D28 of the experiment, three groups of them received a PRRSV vaccination (+V groups) at D0; the 7^th^ group (Control) received a non-contaminated diet and was neither PRRSV infected nor vaccinated against PRRSV. The euthanasia took place over 6 days, designated D44±2.

**Supplementary Table 1:** Feed composition of the two feeding regimens, formulated by DSM-BIOMIN. From D-11 to D13, piglets received a diet named FAF1, thereafter a second diet was provided until the end at D44±2, designated FAF2.

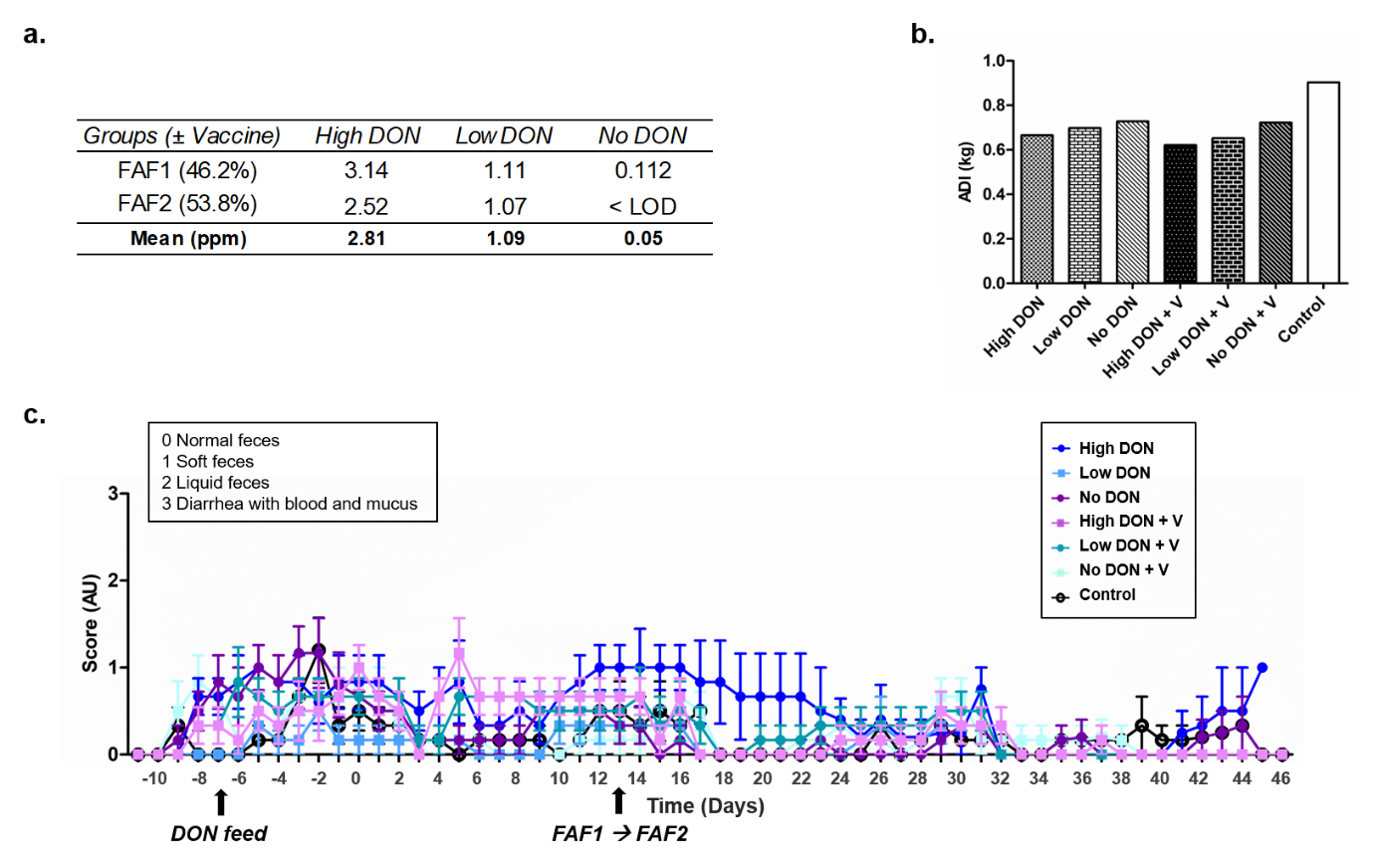


**Supplementary Figure 2:** DON concentration in feed, average daily intake (ADI) and fecal scoring. a) From D-11 to D13, piglets received a first age diet named FAF1 (with or without DON at different concentrations), then a second age diet until the end at D44±2, designated FAF2. The mean of DON exposure corresponds to the concentration of each feed FAF1/ FAF2 taking into account the time that each one was given. Over the entire experiment, pigs in the High DON groups (vaccinated [+ V] or non-vaccinated) received a mean of 2.81 ppm, Low DON groups (vaccinated [+ V] or non-vaccinated) of 1.09 ppm and No DON groups (vaccinated [+ V] or non-vaccinated or Control) of 0.05 ppm. b) The quantities of feed put into feeders and left-overs in the feeders were recorded and used to calculate the average daily intake (ADI) over the entire period of exposure to DON for the different groups, from D-7 to D44±2. c) To assess effect of DON on the intestine, fecal scores of each pig were recorded daily in each group from D-11 to the end (D44±2). 0 = normal feces, 1 = soft feces, 2 = liquid feces and 3 = hemorrhagic feces. The two periods of feed transition influencing the feces score are indicated at D-7 for DON feed and from D13±2 for FAF1 to FAF2.

**Supplementary Table 2: Antibodies used for FCM staining**

| **Antigen** | **Clone** | **Isotype** | **Fluorochrome** | **Labelling strategy** | **Source of primary Ab** |
| --- | --- | --- | --- | --- | --- |
| CD4 | 74-12-4 | IgG2b | Alexa488^a^ | Secondary antibody | In house |
| CD8α | 76-2-11 | IgG2a | PE-Cy7^b^ | Secondary antibody | In house |
| CD8β | PPT23 | IgG1 | Strp-BV421^c^ | Secondary antibody | In house |
| CD27 | b30c7 | IgG1 | Alexa647 ^d^ | Directly conjugated | In house |
| IFN-γ | P2G10 | IgG1 | PE | Directly conjugated | BD Biosciences |
| TNF-α | Mab11 | IgG1 | BV605 | Directly conjugated | BioLegend |

^a^Goat anti-mouse IgG2b-Alexa488, Thermo Fisher, Waltham, MA, USA.

^b^Goat anti-mouse IgG2a-PE-Cy7, Southern Biotech, Birmingham, AL, USA.

^c^Streptavidin Brilliant Violet 421, BioLegend, San Diego, CA, USA.

^d^in house conjugation with Protein Labelling Kit Alexa647, Thermo Fisher.

**
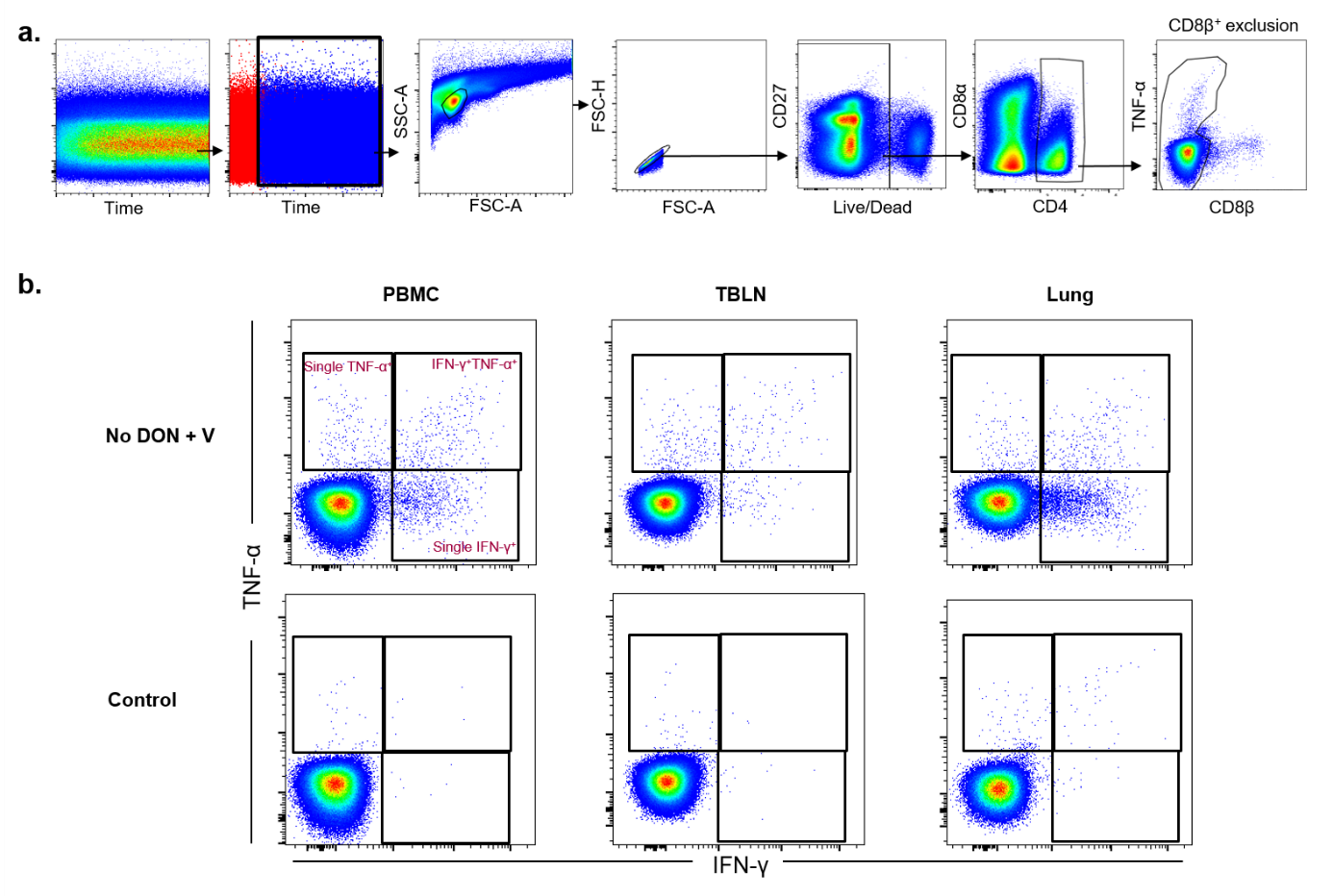
**

**Supplementary Figure 3:** Gating hierarchy and representative flow cytometry data of cytokine producing CD4^+^ T cells following PRRSV re-stimulation *in vitro*. a) After exclusion of the events outside the statistical norm (based on time scale, performed by FlowJo FlowAI algorithm), lymphocytes were gated based on light scatter properties. Thereafter, doublets and dead cells were excluded and CD4^+^ T cells gated. Gating of CD4^+^ T cells was further optimized by the exclusion of contaminating CD8β^+^ cells. b) Total CD4^+^ T cells were gated (as shown in a) and single IFN-γ^+^, single TNF-α^+^, and IFN-γ/TNF-α co-producing cells were gated. Representative data of animals from the No DON + V and the Control group are shown for blood (PBMC), tracheobronchial lymph nodes (TBLN) and lung tissue.

**
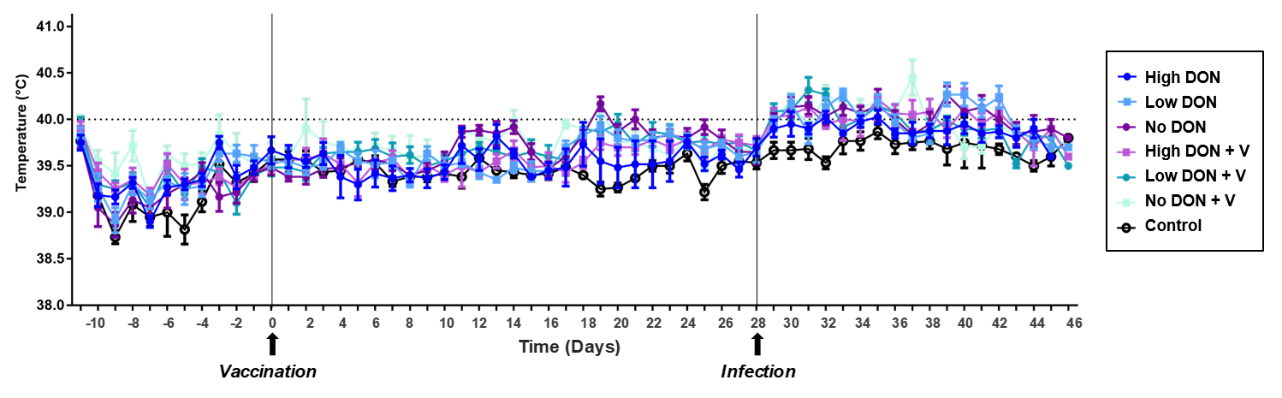
**

**Supplementary Figure 4: Rectal temperatures of pigs for the different groups from arrival to the end of the study.** To assess the effects of PRRSV vaccination and infection, body temperatures of pigs in each group were recorded daily from D-11 to the end D44±2 days. Mean values ± SEM are shown for each group.

**
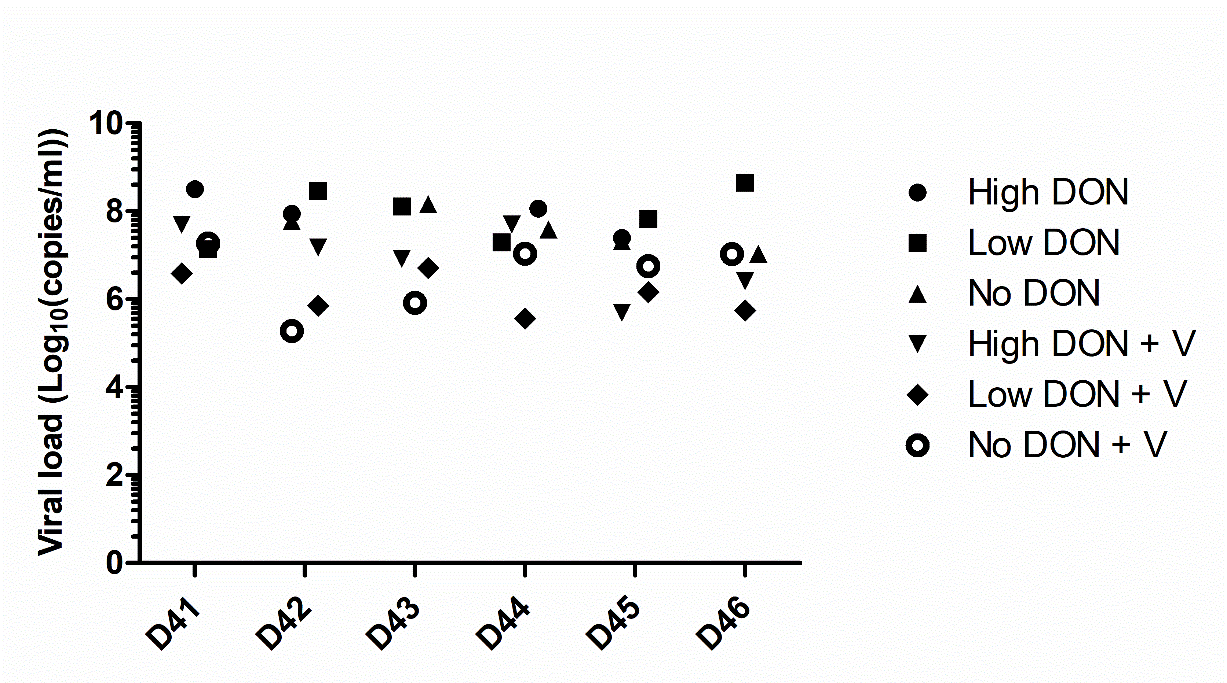
**

**Supplementary Figure 5:** Distribution of lung viral loads over killing days in PRRSV infected pigs. To determine a potential correlation between viral load in lungs and the six necropsy days (D41, D42, D43, D44, D45, D46), an ANOVA test followed by a Bonferroni’s Multiple Comparison Test were applied for all pigs in the study, except the Control group. No influence of the killing day on the level of viral load was found, p<0.05.

**References**

Balka G, Ladinig A, Ritzmann M, Saalmüller A, Gerner W, Käser T, Jakab C et al. (2013) Immunohistochemical characterization of type II pneumocyte proliferation after challenge with type I porcine reproductive and respiratory syndrome virus. J Comp Pathol 149:322-330. https://doi.org/10.1016/j.jcpa.2012.12.006.

Bracarense AP, Lucioli J, Grenier B, Pacheco GD, Moll WD, Schatzmayr G, and Oswald IP (2012) Chronic Ingestion of Deoxynivalenol and Fumonisin, Alone or in Interaction, Induces Morphological and Immunological Changes in the Intestine of Piglets. Br J Nutr 107:1776–1786.

Egli C, Thur B, Liu L, Hofmann MA. (2001) Quantitative TaqMan RT-PCR for the detection and differentiation of European and North American strains of porcine reproductive and respiratory syndrome virus. J Virol Methods. 98:63–75. https://doi.org/10.1016/S0166-0934(01)00358-5

Kreutzmann H, Dürlinger S, Knecht C, Koch M, Cabana M, Torrent G, Balasch M, et al. (2021) Efficacy of a Modified Live Virus Vaccine against Porcine Reproductive and Respiratory Syndrome Virus 1 (PRRSV-1) Administered to 1-Day-Old Piglets in Front of Heterologous PRRSV-1 Challenge. Pathogens 10:1342. https://doi.org/10.3390/pathogens10101342.

Kreutzmann H, Stadler J, Knecht C, Sassu EL, Ruczizka U, Zablotski Y, Vatzia E, et al. (2022) Phenotypic Characterization of a Virulent PRRSV-1 Isolate in a Reproductive Model With and Without Prior Heterologous Modified Live PRRSV-1 Vaccination. Front Vet Sci 9:820233. https://doi.org/10.3389/fvets.2022.820233.

Reutner K, Leitner J, Essler SE, Witter K, Patzl M, Steinberger P, Saalmüller A, and Gerner W (2012) Porcine CD27: Identification, Expression and Functional Aspects in Lymphocyte Subsets in Swine. Dev Comp Immunol 38:321–331. https://doi.org/10.1016/j.dci.2012.06.011.

Sassu EL, Ladinig A, Talker SC, Stadler M, Knecht C, Stein H, Frömbling J, et al. (2017) Frequency of Th17 Cells Correlates with the Presence of Lung Lesions in Pigs Chronically Infected with Actinobacillus Pleuropneumoniae. Vet Res 48:4. https://doi.org/10.1186/s13567-017-0411-z.

Sinn LJ, Zieglowski L, Koinig H, Lamp B, Jansko B, Mosslacher G, Riedel C, Hennig-Pauka I, Rümenapf T (2016) Characterization of Two Austrian Porcine Reproductive and Respiratory Syndrome Virus (PRRSV) Field Isolates Reveals Relationship to East Asian Strains. Vet Res 47:17. https://doi.org/10.1186/s13567-015-0293-x.

Stadler J, Naderer L, Beffort L, Ritzmann M, Emrich D, Hermanns W, Fiebig K, et al. (2018) Safety and Immune Responses after Intradermal Application of Porcilis PRRS in Either the Neck or the Perianal Region. PLoS One 13:e0203560. https://doi.org/10.1371/journal.pone.0203560.

Straw BE, Backstrom L, and Leman AD (1986) Examination of Swine at Slaughter 2. Findings at Slaughter and Their Significance. Comp Cont Educ Pract 8:106–110.
